# Microencapsulation of *Camelina sativa* Oil Using Selected Soluble Fractions of Dietary Fiber as the Wall Material

**DOI:** 10.3390/foods8120681

**Published:** 2019-12-13

**Authors:** Aleksandra Kanclerz, Ewelina Drozińska, Marcin Andrzej Kurek

**Affiliations:** Department of Technique and Food Development, Warsaw University of Life Sciences, 02-787 Warsaw, Poland; kanclerz.aleksandra@gmail.com (A.K.); e.drozinska@gmail.com (E.D.)

**Keywords:** *Camelina sativa*, sea buckthorn oil, microencapsulation

## Abstract

The aim of the study was to prove the usefulness of microencapsulation of *Camelina sativa* oil regarding its vulnerability to oxidation caused by oxygen, temperature, and other factors. Pectin, inulin, gum arabic, and β-glucan, each of them mixed with maltodextrin, were used as wall materials and their appropriability to reduce oxidation of the core material was examined. Microcapsules were prepared by spray drying, which is the most commonly used and very effective method. The research confirmed results known from literature, that gum arabic and inulin are most proper wall materials, because they ensure small oxidation increase during storage (4.59 and 5.92 eq/kg after seven days respectively) and also provide high efficiency of process (83.93% and 91.74%, respectively). Pectin turned out to be the least appropriate polysaccharide because it is not able to assure sufficient protection for the core material, in this case *Camelina sativa* oil, due to low efficiency (61.36%) and high oxidation (16.11 eq/kg after seven days). β-glucan occurred to be the coating material with relatively high encapsulation efficiency (79.26%) but high humidity (4.97%) which could negatively influence the storage of microcapsules. The use of polysaccharides in microencapsulation, except performing the role of wall material, has the advantage of increasing the amount of dietary fiber in human diet.

## 1. Introduction

Microencapsulation is the process of creating a functional barrier between the core and shell materials to avoid chemical or physical reactions and to maintain the biological and physicochemical properties of the core material. This is done by surrounding molecules or drops of the coating material or embedding them in a homogeneous or heterogeneous matrix to form capsules. This allows the movement of a solid, liquid or gas substance through another substance in the form of a capsule [1].

The dynamic development of food and nutrition sciences, dictated by the increasing demands placed by the market and consumers, makes it necessary to use technology that will allow for the introduction of food components that have beneficial effects on health while maintaining their functionality and bioavailability. In many cases, microencapsulation may be an appropriate way to protect these components, for example, against oxidation processes [2].

The coating material is defined as a layer of a substance that forms a coating around the core to form microcapsules. The coating material may be a natural or synthetic polymer, which is selected based on the predetermined capsule characteristics. The composition of the coating is the primary determinant of the functional properties of the capsules and a way to improve the encapsulation efficiency of a given component [2,3]. The coating material should have the following features: water solubility, chemical core fit, film formation ability, non-hygroscopicity, elasticity, stability, flexibility, low viscosity, economy, and ability to gradually release the core [3].

Camelina oil is extracted from the seeds of *Camelina sativa*, a plant belonging to the *Brassicaceae* family. Camelina seeds contain about 30–40% oil. Camelina oil is rich in polyunsaturated fatty acids, among which linolenic acid and α-linolenic acid are mainly notable as good sources of omega-3 fatty acids. Camelina oil, thanks to its composition, has beneficial effects on human health [4]. The oil is mainly composed of polyunsaturated fatty acids in an amount of 55.38% according to O’Dwyer et al. [5], while Abramovič and Abram [4] consider the amount to be 55.8%, among which the most abundant is α-linolenic acid (335–40% according to Belayneh et al. [6], 35.2% according to Abramovič and Abram [4], and 33.8% according to O’Dwyer et al. [5]). The oil is characterized by a low content of erucic acid, which is considered to be toxic to humans, because it causes, among other things, the steatosis of internal organs and damages the myocardium, so its content is a determinant of the possibility of using oil in human nutrition. It should not exceed 5% [4].

As a coating material in the microencapsulation, polysaccharides are often used. Maltodextrin is one of the most common polysaccharides that is employed in microencapsulation processes due to its low viscosity at high solids concentration, good solubility, good protection against oxidation, relatively low cost, and neutral aroma. However, maltodextrin could not be used alone due to poor emulsifying capacity and emulsion stability. Therefore, other polysaccharides could be added to maltodextrin to act as wall material in microencapsulation. From which many of them are classified as dietary fiber. The soluble fiber fraction consists of β-glucans, fructooligosaccharides, pectins, gums and plant sluices, and some hemicelluloses. It is soluble in water and can produce gels. It reduces the absorption of cholesterol from food and contributes to lowering the level of LDL cholesterol in the blood. It is also used to treat constipation and provides a smaller insulin response after a meal. β-glucans also show immunomodulatory effects, enhancing the action of immune cells and accelerating the production of blood in the bone marrow. Sources of soluble fiber are cereals, mainly oats and barley, fruits and vegetables, nuts, flax seeds, seeds of plantago psyllium and leguminous plants [3,7].

There are several different methods of achieving microcapsules like spray-drying, coacervation, extrusion, liposomal encapsulation etc. However, spray drying is perceived as the method with low cost and high efficiency as well as application to a wide range of products. Another advantage of spray drying is the rapid solubility of capsules, the small size of particles and their high durability [3].

So far, no comprehensive study describing the ability to form microcapsules has been found in the literature wherein the core material is Camelina sativa oil and the coating material is polysaccharide, which is classified as soluble dietary fiber fraction. Therefore, in the presented study, the usefulness of selected polysaccharides, categorized as dietary fiber fractions as a coating material, was evaluated to preserve Camelina sativa oil properties and reduce oxidation. The evaluation was carried out using the most common microencapsulation method—spray drying.

## 2. Materials and Methods

### 2.1. Materials

The oil used for the study was attended with the certification of organic farming to prevent contamination with other oils (Camelina oil pressed cold, Gifts of Nature, Poland, Certificate No.: PL-EKO-01-001493). Pectin, inulin, maltodextrin with a glucose equivalent of DE 18-20, and gum arabic were originated from Agnex firm, in one delivery (Białystok, Poland). Pectin was extracted from apples, and inulin was extracted from chicory. β-glucan used in the studies was extracted from barley by the method proposed by Kurek et al. [8] and had a concentration of 85%.

### 2.2. Preparation of Emulsions and Microcapsules

To prepare the microcapsules, the coating material was added into distilled water and stirred on a magnetic stirrer. The emulsions were prepared in a ratio of 80:20 (*v*/*v*) with the previously prepared aqueous solution in oil (20%). In the aqueous solution, the concentration of solids was maintained at 30% in the ratio maltodextrin: dietary fiber 9:1. This concentration was assessed as the most effective in terms of drying with maintaining viscosity allowing it to be pumped through the peristaltic pump and nozzle of spray-dryer after preliminary study. The emulsification process was done with a rotor-stator homogenizer (Ultra-Turrax, IKA, Staufen, Germany) at 16,000 rpm for 5 min. Each emulsion was prepared with three repetitions. The emulsions were stirred on magnetic stirrer throughout the spray drying process to obtain the microcapsules. The spray-dryer Buchi B-290 (Büchi, Flawil, Switzerland) was used with the inlet temperature 160 °C and outlet temperature 80 °C with peristaltic pump 7.5 mL/min and air flor 600 L/h. The emulsion was atomized using the nozzle with a diameter of 1.5 mm.

### 2.3. Emulsions Stability

Emulsions stability was based on the following formula:

(1) $$CI\;\left(\%\right)=\;\frac{H_{s}}{H_{e}}\times 100$$

where *CI* (%)—gelling rate, *H_s_*—sediment layer height, *H_e_*—total height of emulsion.

Immediately after preparation, the emulsion (O/W) was poured into glass tubes of 88 mm and 25 mm diameter with plastic caps to prevent evaporation. They were left for 2 h to stabilize followed by *H_s_* and *H_e_* measurements. Each emulsion was tested with three repetitions.

### 2.4. Measurement of Emulsion Particle Size

The particle size measurement of the emulsion was carried out using Morphologi^®^ G3SE (Malvern Instruments Ltd., Malvern, UK) equipped with a system for determining the particle size of liquid substances. The measurement method was based on Desplanques et al. [9] and Piwińska et al. [10] with minor modifications.

1 mL of the sample was diluted in a 30 mL solution of 0.5% SDS (sodium dodecyl sulfate) and stirred for 5 min at 300 rpm on a magnetic stirrer (SI Analytics GmbH, Mainz, Germany). The samples were then allowed to stand for 10 min to get rid of possible air bubbles. The measurements were carried out with three repetitions. Four parameters were chosen to describe the size of the emulsion droplets: the average diameter D (*N*, 4.3) of all particles (μm), the size of the smallest particles D (n 0.1) (μm), the average particle size D (*n* 0.5) (μm), and the largest particle size D (*n* 0.9) (μm), corresponding to 10%, 50% and 90% of particles under the reported particle size. All parameters were automatically calculated during the measurement.

### 2.5. Density and Humidity of Microcapsules

Each of the prepared powders was applied to a graduated cylinder, after which the volume in the cylinder was read and weighed. Density was expressed as a ratio of mass to volume (g/cm^3^). Each powder was tested with three repetitions.

The moisture content of microcapsules was measured using an RAD WAG MA 50 moisture analyzer and was expressed as a percentage. Each powder was tested with three repetitions.

### 2.6. Colorimetric Measurement

The color of the capsules produced was measured using a Minolta CR-400 colorimeter (Konica Minolta Inc., Tokyo, Japan) with the parameters: illuminant D65, measuring surface ø = 8 mm, standard 2° observers. The results are expressed according to the CIELab color space. The specified parameters were *L* * (*L* = 0 − black, *L* = 100 − white), *a* * (−*a* = green, +*a* = red), and *b* * (−*b* = blue, +*b* = yellow). Each powder was tested with three repetitions.

### 2.7. The Particle Size of the Microcapsules

The particle size distribution was determined using a Morphologi^®^ G3SE apparatus (Malvern Instruments Ltd., Malvern, UK) equipped with a dispersion unit for dry samples. Each powder was tested with three repetitions. The particle size distribution was calculated as the relative volume of particles in the size bands shown as size distribution curves (Malvern Microsoft ware v. 5.40, Malvern Instruments Ltd., Malvern, United Kingdom). To describe the particle size distribution, the following parameters were selected: size of the largest particles (D [*v*, 0.9]), size of the average particles (D [*v*, 0.5]), and size of the smallest particles (D [*v*, 0.1]) corresponding to 10%, 50% and 90% of particles under the reported particle size. The span was estimated with the following formula:

(2) $$Span=\;\frac{\left(D_{0.9}-D_{0.1}\right)}{D_{0.5}},$$

where D_10_ is the size of the smallest particles, D_50_ the size of the average particles, and D_90_ is the size of the largest particles.

### 2.8. Determination of Microencapsulation Efficiency

Microencapsulation efficiency was estimated using the following formula:

(3) $$EE\;\left(\%\right)=\;\frac{T_{o}-S_{o}}{T_{o}},$$

where *T_o_* is total oil and *S_o_* is surface oil.

#### 2.8.1. Measurement of Surface Oil

The dried microcapsules, in an amount of 0.5 g, were dispersed in 5 mL of hexane and then stirred for 30 s at room temperature. They were then filtered through anhydrous sodium sulfate. The solvent was evaporated using a rotary evaporator at 40 °C (R 100 Büchi, Flawil, Switzerland) until a constant weight was obtained. Each type of microcapsules was tested with three repetitions.

#### 2.8.2. Measurement of Total Oil

In the falcon of 50 mL, 1 g of prepared microcapsule was weighed, 25 mL of isooctane: isopropanol 2:1 mixture was added, followed by vortexing (Vortex Mixer MX-S, DLAB, Riverside, California, USA) for 30 s. Shaking was then carried out on the rotor for 15 min (Intelli Mixer RM-2, Elmi Ltd., City, Latvia) with a vibrating program, after which 1 mL of distilled water was added. The extract tubes were centrifuged for 10 min, and then the upper organic phase was collected and passed through a filter with anhydrous sodium sulfate to a pre-weighed flask of 25 mL. The solvent was evaporated on a rotary evaporator at 45 °C (R-100 Büchi, Flawil, Switzerland) until a constant weight was obtained. The difference between the weight of the dry flask and the flask with the residue after evaporation was the total oil content. Each type of microcapsules was tested with three repetitions.

### 2.9. Fatty Acids Profile

Obtained fat samples, according to the methodology for determining total oil, were analyzed for the fatty acid profile. The flask was accurately weighed, and the residue was dissolved in such an amount of hexane that 100 mg corresponded to 1 mL of hexane. 1 mL of hexane was withdrawn into a twisted glass bottle, then 1 mL of 1 M KOH in methanol was added and allowed to stand in a gentle shaking water bath for 6 min at 55 °C. Then 1 mL of distilled water, 0.5 mL of saturated brine, and 1 mL of hexane were added and mixed. The phase separation was carried out for 30 min, after which the clear organic layer was transferred to an autosampler vial. The measurement was carried out using a gas chromatograph according to the methodology of Kurek et al. [8]. Each type of microcapsules was tested with three repetitions.

### 2.10. Peroxidase Value

Dried microcapsules weighing 0.25 g were dispersed in 2.5 mL of distilled water in a test tube. The tube was then shaken for 30 min to dissolve the powder. After this time, 300 μL were withdrawn from the tube, 1.5 mL isooctane: isopropanol 2:1 was added and vortexed three times for 10 s to ensure oil extraction. The phases were then separated by centrifugation (Hettich Universal 320R, Tuttlingen, Germany) (1000× *g* for 4 min). After centrifugation, 200 μL were taken to determine the peroxidase value.

Peroxide number based on spectrophotometric measurement, using the iron oxidation changes according to the methodology of Shantha and Decker [11], was used to determine the lipid oxidation. Each type of microcapsules was tested with three repetitions. Peroxidase value was assessed after one and seven days after spray-drying and storing in 25 °C.

### 2.11. SEM Analysis

For SEM analysis, small amounts of the sample were placed on the surface of the double-sided tape attached to the tips. The samples were then observed under vacuum using a QUANTA 200 scanning electron microscope. Each type of microcapsules was viewed at an enlargement of 5000 and 10,000 times. Each type of microcapsules was tested with three repetitions.

### 2.12. Statistical Analysis

The obtained results were subjected to statistical analysis for the study of one-way variance followed by Tukey’s tests and for the results, means were compared by the least significant difference (LSD) employing Statistica 13 (Statsoft, Tulsa, Oklahoma, USA). *p* values ≤ 0.05 were considered as significant and each measurement was triplicated.

## 3. Results and Discussion

### 3.1. Emulsion Stability

The results of stability of the emulsions produced are shown in Table 1. It was expressed as the height of the suspended sediment layer with the total height of the emulsion in the test tube. Pectin was the most stable (84.11%), the emulsions of inulin and gum arabic were characterized by comparable stability (75.93% and 75.00% respectively), while β-glucan was characterized by the lowest stability (30.00%), and this result is significantly different from the other three. The term, emulsion stability, means the ability to retain properties over a given period of time. Considering that the emulsions are thermodynamically unstable, changing their property over time is inevitable, and the slower these changes take place, the more stable the emulsion is. Phenomena that may affect the stability of emulsions include coalescence, flocculation, and creaming [12]. According to Temelli [13], in the above-mentioned study, the highest obtained stability of β-glucan emulsion was 63%, which is much higher than the result obtained in this study. The stability of the emulsion was tested in 4 temperature variants and four variants of pH, and it showed that higher temperature and low pH favor higher emulsion stability (in this case, the highest stability was achieved with a combination of 55 °C and pH 7.0). Differences between the obtained results may be due to different study conditions as the emulsions in this study were at room temperature, which may affect their stability, or followed different methods of β-glucan extraction. However, due to the lack of available research in this area, it is difficult to relate and compare the obtained result to other literature results. It can be assumed that in the case of the β-glucan emulsion, there was a flocculation phenomenon, which means merging emulsion particles into larger aggregates, causing them to precipitate from the emulsion in the form of sediment or turbid suspension, which significantly reduced the degree of stability of this emulsion. According to Böger et al. [14], in the emulsion containing gum arabic and maltodextrin as the coating material, the phase separation occurred, whereas, in the emulsion containing only gum arabic, this phenomenon did not occur. This may be due to the lack of emulsifying properties exhibited by maltodextrin. The high viscosity of the emulsion, due to the properties of the polysaccharides used or their high concentration, can reduce the emulsion sedimentation coefficient, thereby increasing stability [15].

### 3.2. Measurement of Emulsion Particle Size

The results of particle size of the emulsions are shown in Table 1. According to Böger et al. [14], it is desirable to have the smallest particle size for the emulsion as they yield better microencapsulation efficiency. The largest molecules of the emulsion were observed in β-glucan (44.85 μm) and gum arabic (44.84 μm) emulsions whose particles were almost similar in size. In the case of these two emulsions, the possibility of coalescence should be considered, which means combining the particles of the dispersed emulsion phase into larger particles. This could explain the much larger particle size of these emulsions compared to the other two. Smaller particles had inulin (18.83 μm), while the smallest ones were pectin emulsion (14.67 μm). According to Böger et al. [14], reducing the amount of shell-active material, in this case—gum Arabic, enables covering the oil drop completely, increasing the particle size of the emulsion. However, it reduces the viscosity of the solution, which can reduce the particle size.

### 3.3. Density and Humidity of Microcapsules

The results of density of the microcapsules are shown in Table 1. It is expressed in g/cm^3^. Gum arabic microcapsules (0.554 g/cm^3^) were the densest, followed by β-glucan (0.553 g/cm^3^) and pectin (0.517 g/cm^3^), and all of them were in the same statistical group, which indicates the fact that the differences between them are very nominal and statistically insignificant. The lowest density was found in inulin capsules (0.367 g/cm^3^). According to Ahmad et al. [16], the density of the microcapsules in the form of a powder is related to the molecular weight of the material as the material with a larger mass readily occupies the free spaces between the molecules, leading to a reduction in the overall volume of the powder and thus an increase in density. In studies carried out by Böger et al. [14], capsules with gum arabic as a coating material had a density of 0.40 g/cm^3^, which is lower than the result obtained in this study and is convergent with the size of the emulsion particles obtained by Böger et al. [14], which were also smaller than the result obtained in the study. This result is also similar in the studies of Fernandes et al. [17] who obtained capsules of gum arabic with a density of 0.41 g/cm^3^.

The moisture content of the microcapsules is shown in Table 1. The highest moisture content was found in microcapsules from β-glucan (4.97%), then from gum arabic (3.54%), while the smallest one was from pectin (3.04%) and inulin (2.80%), located in the same statistical group. Polysaccharide dietary fiber components are strongly hydrophilic, which causes water to be added to them and retained in empty spaces in the molecular structure [7]. β-glucan has the most excellent feature to maintain a significant amount of water due to its crosslinked structure and hydrophilic character; the water retained in the β-glucan network is heavily released from it, which may increase the humidity of the capsules [18]. Differing results were obtained by Fernandes et al. [17] because, in their studies, capsules made of gum arabic had a humidity of 1.64%. The moisture content of the capsules is related to the properties of the coating materials and the speed at which they form a shell around the core during spray-drying. The faster this process takes place, the higher the humidity of the capsule, because after the coating has formed quickly, the water formed diffuses and evaporates from its interior. Therefore, it is essential to choose the right coating material and drying process parameters, as well as the proportion of ingredients in the emulsion, because a more substantial amount of coating material in the emulsion may cause faster coating around the core, which makes it difficult for water to evaporate and increase humidity [19].

### 3.4. Colorimetric Measurement

The results of the color measurement are presented in Table 1. The brightness of the capsules, described by the L* value with a conventional range from 0 to 100 (from black to white), ranged from 86.7 to 93.7. The color of the materials themselves determined the differences in the color between the capsules with different coating materials. Regardless of the type of capsule, the core material (camelina oil) remained always the same. Due to its intense color, it can be assumed that the color of the capsules deviated from the color of the raw coating materials. The differences also resulted from the fact that each coating material has different properties and creates a coating of different thickness around the core. The capsules of the highest intensity of white were made of inulin (they had the brightest color), while β-glucan capsules represented the smallest intensity of whiteness (the darkest color). It is a statistically significant difference. However, the difference in color was not noticeable without colorimetric analysis.

The *a* * parameter described the red-green relation, where positive values indicated red and negative values indicated green. All examined capsules were characterized by negative values of the *a* * parameter, which means that they had a color closer to green. The greenest and the least red (the smallest *a* * value) were characterized by inulin capsules, while the least green ones (with the highest *a* * value) were pectin capsules. The *b* * parameter describes the yellow-blue relationship, where positive values indicated yellow and negative values indicated blue. All examined capsules were characterized by positive values of parameter *b* *, which means that they had a color closer to yellow. The most yellow and least blue (with the highest *b* * value) had β-glucan capsules, while the least yellow (with the smallest *b* * value) was inulin capsules. In studies by Kurek et al. [20], pure β-glucan extracted from barley had the parameters *L* * = 79.69, *a* * = 3.41, *b* * = 13.57. It is a result that differs from the one obtained in this study, but it only concerns β-glucan and not microcapsules possessing core material, which could also affect their color.

### 3.5. The Particle Size of the Microcapsules

The polydispersity index of the microcapsules are shown in Table 1. It indicates the statistical dispersion of polymer molecules. The smaller it is, the more similar in size the molecules are.

The largest overall capsule diameters were with inulin (16.734 μm) as the coating material, while the smallest ones with β-glucan (10.292 μm). The smallest polydispersity index (0.806) was presented by capsules made of pectin, which means that they had molecules of the least diversified size. The largest particle size distribution was observed in the case of β-glucan capsules (1.090). The particle size may be related to viscosity since molecules with higher molecular weights tend to have higher viscosities [16], and as reported by Fernandes et al. [17], the higher the viscosity of the coating material used, the larger the capsule.

In studies by Fernandes et al. [21] concerning the microencapsulation of rose oil with arabic gum, the capsules had a total diameter of 13.6 μm, and the D10, D50, and D90 values were successively 3.12 μm, 10.75 μm, and 26.06 μm, which indicates a much larger particle size distribution described by the polydispersity index (*Span* = 2.13) than that obtained in this study (*Span* = 1.012). The overall diameter of the particles has similar value as in this study (from 14.67 µm for pectin microcapsule to 44.85 for β-glucan sample). Böger et al. [14] obtained microcapsules from a mixture of gum arabic and maltodextrin as a coating material with an overall diameter of 26.96 ± 0.46 μm. This is a result that significantly exceeds even the largest capsules with this coating material obtained in the study. According to Fernandes et al. [17], the size of the capsules produced during spraydrying depends on atomization, the properties of the coating material, its concentration and viscosity, and the conditions of the drying process.

### 3.6. Microencapsulation Efficiency

The effectiveness of microencapsulation is presented in Table 1. This is one of the most important parameters to describe the microencapsulation process because it determines the ability of the coating material to encapsulate the core material. The amount of surface and total oil in the microcapsule were considered for determining the efficiency. The higher the amount of surface oil, the lower the efficiency, as the goal of the whole process is to close as much of the core material inside the microcapsule as possible [16]. The highest efficiency of microencapsulation occurred in the case of inulin capsules (91.74%), which means that they had the least surface oil compared to the whole oil of the capsule, while the smallest in the case of pectin capsules (61.36%), which had the most significant amount of oil surface. Similar efficiency was presented by gum arabic capsules (83.93%) and β-glucan capsules (79.26%), which were in the same statistical group.

In the study of Ahmad et al. [16], the efficiency of microencapsulation in the β-glucan coating was only 45%. As reported by Böger et al. [14] after Jafari et al. [22], small drops of oil in the initial emulsion will be more efficiently encapsulated in the coating material and also provide better emulsion stability during spray drying. The larger the size of the emulsion particles, the greater their disintegration during atomization in the chamber during spray drying, which changes the particle size distribution. This disintegration increases the amount of surface oil, which reduces the efficiency of microencapsulation and also increases the oxidation of the oil. Efficiency can, therefore, be increased by decreasing the particle size of the emulsion. The different information is presented by Fernandes et al. [17], stating that the increase in the efficiency is affected by the rise in the size of microcapsules because with the increase in particle size, its surface to volume ratio decreases, so the amount of total oil relative to the surface volume also increases. This is confirmed with this research.

In the study of Böger et al. [14], the microencapsulation of the emulsion with a mixture of maltodextrin and gum arabic (as in the own study) had an effectiveness of 63.47 ± 0.49%. In our research, the particles were much larger than those obtained by Böger et al. [14], yet the efficiency of microencapsulation is higher, which is in contradiction with the assumptions adopted by them. This may be the result of an incorrectly chosen inlet temperature during the spray drying process. As reported by Chew et al. [19], the efficiency of microencapsulation can be increased by increasing the amount of coating material in the emulsion, because during spray drying, the coating around the core is produced more efficiently and more quickly, preventing it from depositing on the surface of the capsule. In studies by Li et al. [15], a high concentration of solids resulted in less deposition of oil on the surface of the capsule, which increased efficiency. However, too high a concentration of polysaccharides in the emulsion can significantly increase the viscosity of the solution, which will interfere with the spraying of the emulsion during drying and lead to the irregular shape of the capsules.

### 3.7. Fatty Acids Profile

The profile of selected fatty acids of the oil is presented in Table 2. It was determined based on gas chromatography. The camelina oil was characterized by the highest amount of polyunsaturated fatty acids, followed by monounsaturated fatty acids, while it was characterized by the lowest content of saturated fatty acids, which is confirmed in the literature. The main polyunsaturated acids were α-linolenic acid (34.73%) and linolenic acid (18.07%); the main monounsaturated fatty acids were oleic acid (15.58%) and *cis*-11-eicosanoic acid (14.31%), and the saturated one was palmitic acid (5.23%). Tiny amounts of omega-9 monounsaturated fatty acids were also detected: erucic (3.12%) and neuronal (1.21%). The low erucic acid content is particularly crucial for edible oils due to its toxic effects on humans.

This profile of fatty acid of camelina oil is in consonance with what has been found by O’Dwyer et al. [5], in which research was detected higher content of palmitic acid (5.61%), stearic (2.49%), and oleic acid (16.37%) and lower content of α-linolenic acid (33.80%), linolenic (17.95%), and eicosanoic (14.24%). Abramovič and Abram [4] marked a larger amount of α-linolenic acid (35.20%), palmitic acid (6.43%), and oleic acid (17.40%) and a lower amount of stearic acid (2.57%), linolenic acid (16.90%), and erucic (1.62%). Eidhin et al. [23] marked a higher content of palmitic acid (5.50%), α-linolenic acid (38.90%), and eicosanoic acid (16.20%), and lower content of erucic acid (2.40%), oleic acid (14.90%), and linolenic acid (15.8%). The most substantial differences were found between the content of oleic acid (O’Dwyer et al. [5] found 16.37%, Abramovič and Abram [4] found 17.40%), but these are not statistically significant differences. The profile of selected fatty acids in this study and literature studies is very similar.

The fatty acid profile is different in each type of microcapsules and in pure camelina oil. The smallest differences concern eicosan fatty acid (from 14.31% to 14.54%), erucic fatty acid (from 3.12% to 3.21%), and stearic fatty acid (from 2.38% to 2.40%). Each type of used dietary fiber has different molecular weight and therefore could form walls with diverse thickness. This could influence the fatty acids profile because not all of the samples had the same contact with hot air during spray-drying even they deposit in the dryer for the same amount of time. This could lead to the degradation of fatty acids—especially PUFA and MUFA during spray-drying—and their hydrolysis [24].

### 3.8. Peroxidase Value

**Figure 1:**
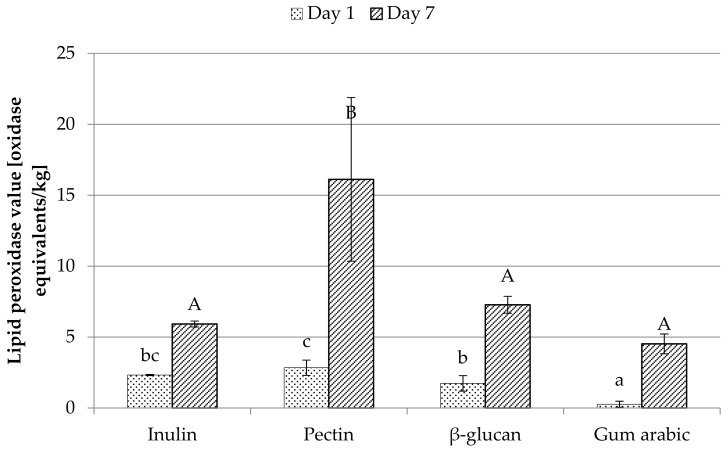
Lipid peroxidase value on the day of preparing microcapsules (day 1) and on the seventh day. Letters a, b, c, d refer to the measurement on the first day, and A, B, C refer to the measurement on the seventh day and indicate homogeneous groups statistically, with the level of significance *p* ≤ 0.05.

Comparison of lipid peroxidase value on the day of microcapsule preparation and on the seventh day is presented in Figure 1. The degree of oxidation of lipids was expressed in oxidation equivalents/kg of oil. A substantial increase in the oxidation state of the unencapsulated oil can be observed (from 4.28 on the first day to 41.02 on the seventh day) compared to the one that was encapsulated. The highest degree of oxidation was detected in pectin capsules (2.84 on the first day, 16.12 on the seventh day), while the smallest was in the arabic gum microcapsules (consecutively 0.26 and 4.52). This means that the degree of oxidation of lipids encapsulated with arabic gum after a week of storage is slightly higher than that of pure, non-capsulated oil on the first day. Böger et al. [14] showed in their study that capsules with a mixture of maltodextrin and gum arabic as a coating material protect the oil better from oxidation than those having only gum arabic. They argued with the hydrophilic nature of maltodextrin, which could reduce the oxygen permeability (as a hydrophobic substance) by coating the microcapsule, which reduced the exposure of the oil to oxygen. They obtained the degree of lipid oxidation at the level of 22.5 ± 0.1 equivalent to oxidation/kg of oil, on the day of microcapsule production, which is a result significantly higher than that obtained in this study. In their study, grape seed oil was used, which is characterized by a similar structure of fatty acids (similar amounts of polyunsaturated, monounsaturated, and saturated fatty acids). However, the amount of specific fatty acids is different, hence a large difference may be obtained compared to the results obtained in this study. This may also be determined by unfavorable oil storage conditions. The lower efficiency of encapsulation obtained by them should also be taken into account, which means that capsules prepared by them had larger amount of surface oil, directly exposed to oxidation by contact with oxygen and temperature. Efficiency could be a decisive factor, conditioning a high degree of oxidation. According to Bakry et al. [1], due to high temperatures in the spray drying process, microencapsulated oil is exposed to oxidation. The oxidation of the encapsulated oil can also be reduced by selecting the right mixture of coating materials, as one material might not have all the features that make it the most suitable. For example, in a study by Chew et al. [19], in the coating material, consisting of gum Arabic and sodium caseinate, β-cyclodextrin was added to fill the voids between these materials and reduced the access of oxygen in the oil encapsulated. In studies by Belayneh et al. [6], control test of camelina oil began to show changes in oxidation at day 16, while emulsions made with wheat protein isolate only on day 28, which is explained by the surrounding of oil drops by the protein isolate. This indicates the need to protect the oil from oxidation. In a study by Eidhin et al. [23], in which the oxidation status of various vegetable oils stored at 65 °C for 16 days was checked, the camelina oil, on day 16, was characterized by a lower degree of oxidation than fish, linseed, corn, sesame, and sunflower oil.

### 3.9. SEM Analysis

**Figure 2:**
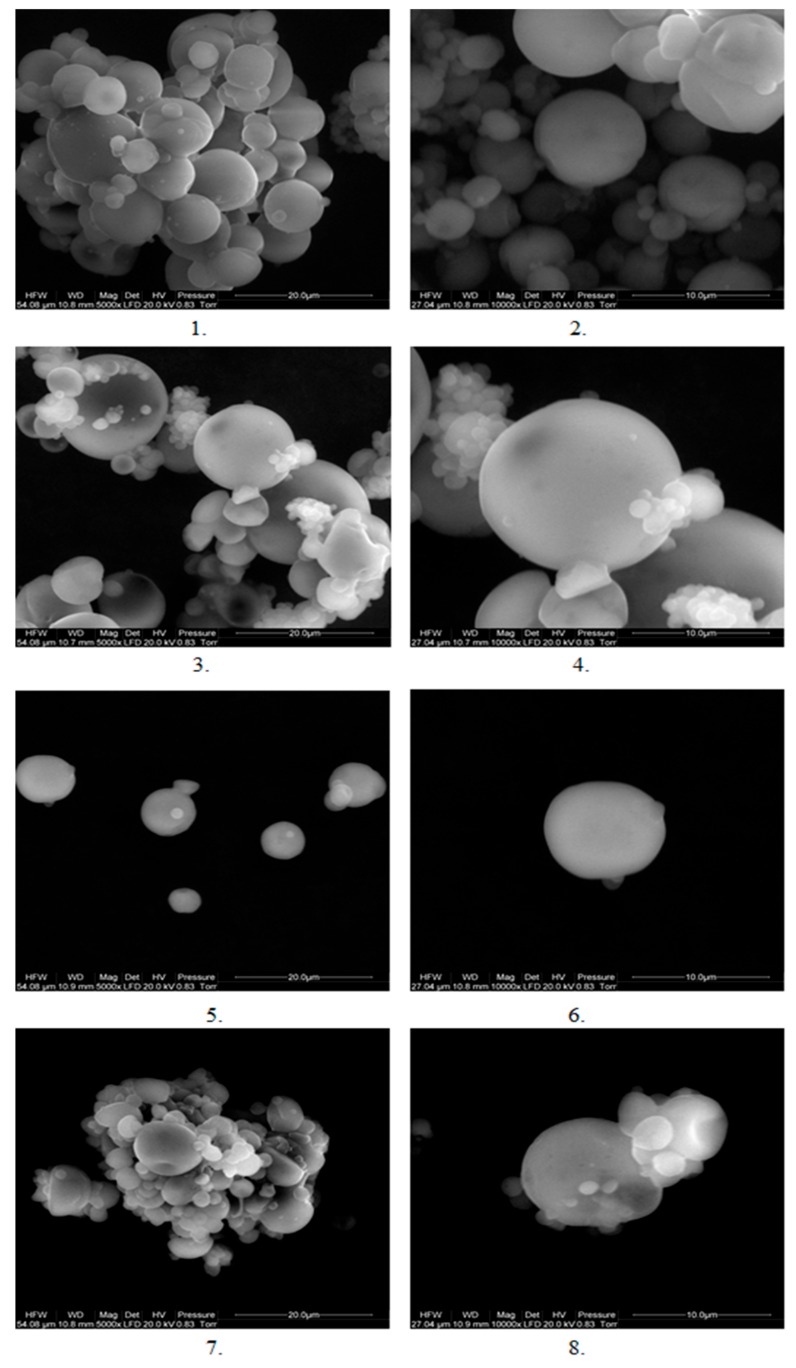
Image of microcapsules under the scanning electron microscope, where **1**—inulin capsules at an enlarged 5000 times, **2**—inoculums 10,000 times magnified, **3**—pectin capsules magnified 5000 times, **4**—pectin capsules 10,000 times magnified, **5**—β-glucan capsules magnified at 5000 times, **6**—glucan capsules at a magnification of 10,000 times, **7**—Arabic gum capsules in a magnification of 5000 times, **8**—capsules of Arabic gum at a magnification of 10,000 times.

Microcapsules with each type of coating material were determined using a scanning electron microscope at two magnifications: 5000 times and 10,000 times. The result of the study is presented in Figure 2. As it can be seen in the microscope image, all types of capsules were characterized by a shape similar to the sphere. From the image, it can be said that the least aggregated capsules were those made of β-glucan (5 and 6), which were captured as single particles, while the other capsules were aggregated to a much greater extent, which made it difficult to capture in a single image capsule. In the picture, the heterogeneity of the size of the capsules is noticeable, especially in the case of pectin (3 and 4) and gum arabic (7 and 8). In the case of these two types of capsules, we observed the attachment of smaller particles to larger ones and the creation of large aggregates. As reported by Böger et al. [14], microcapsules produced by spray drying are characterized by irregular shape and size, which is confirmed in this study. Capsules, however, are not cracked, which may be good protection against oxidation. But the irregular shape and cavities increased the surface of the capsules, which may increase their susceptibility to oxidation [14]. The formation of cavities was caused by the formation of air bubbles inside the capsules after the envelope was formed around them, which caused the capsules to bulge and sink when the temperature exceeded the boiling point of water [25]. According to Chew et al. [19], the recesses on the surface of the capsules can be caused by a large amount of coating material that forms an irregular surface of the coating.

## 4. Conclusions

Capsules made of gum arabic were characterized by the lowest level of lipid oxidation on both measurement days while maintaining high efficiency, which confirmed its validity in the conducted research. The highest efficiency of microencapsulation and, at the same time, lowest lipid oxidation in the measurements performed on both days (one and seven) showed capsules having inulin as a coating material. These two parameters make it a suitable material for coating the oil. β-glucan, due to its unique pro-health properties, is a promising coating material that can be used in the microencapsulation process. Due to the lack of available research in the field of its use as a coating material, it seems reasonable to carry out research aimed at increasing its applicability. The most inadequate coating material for microencapsulation of the oil turned out to be pectin because capsules made from it had the lowest efficiency, and the oil contained in them oxidized to the greatest extent within seven days of storage. Combining it with other coating material to increase its applicability should be considered. The use of soluble fiber fractions as coating materials in the microencapsulation of the camelina oil allows not only to protect the oil against oxidation but also to increase the amount of fiber in the products in which the capsules will be applied. This is a desirable phenomenon as fiber intake has pro-health and preventive effects for many diseases. This study revealed that there could be differences in the properties of microcapsules even if the wall material is composed of the sample with similar chemical structure like polysaccharides. There is still a need to perform more research on how the quality of highly nutritive and sensitive oil could be maintained.

## Figures and Tables

**Table 1:** Properties of emulsions and microcapsules, colorimetric data of microcapsules and span.

	Emulsion Stability [%]	Emulsion Particle Size [µm]	Microcapsules Density [g/cm^3^]	Microcapsules Humidity [%]	Microcapsulation Efficiency [%]	*L* *	*a* *	*b* *	Span	Color Bar
Inulin	75.93 ± 0.21 ^b^	18.83 ± 0.11 ^b^	0.367 ± 0.012 ^a^	2.83 ± 0.12 ^a^	91.74 ± 0.12 ^c^	93.657 ± 0.244 ^d^	−1.927 ± 0.074 ^a^	7.420 ± 0.300 ^a^	0.972 ± 0.019 ^b^	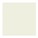
Pectin	84.11 ± 0.19 ^c^	14.67 ± 0.19 ^a^	0.517 ± 0.009 ^b^	3.04 ± 0.17 ^a^	61.36 ± 0.24 ^a^	89.177 ± 0.390 ^c^	−1.383 ± 0.047d ^c^	8.477 ± 0.095 ^b^	0.806 ± 0.012 ^a^	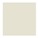
β-glucan	30.00 ± 0.13 ^a^	44.85 ± 0.21 ^c^	0.553 ± 0.013 ^b^	4.97 ± 0.09 ^c^	79.26 ± 0.11 ^b^	86.700 ± 0.130 ^a^	−1.860 ± 0.108 ^a^	9.437 ± 0.534 ^c^	1.090 ± 0.022 ^d^	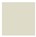
Gum arabic	75.00 ± 0.11 ^b^	44.84 ± 0.12 ^c^	0.554 ± 0.003 ^b^	3.54 ± 0.11 ^b^	83.93 ± 0.09 ^b^	87.923 ± 0.191 ^b^	−1.663 ± 0.050 ^b^	7.447 ± 0.172 ^a^	1.012 ± 0.029 ^c^	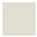

^a, b, c^—letters mean significantly different results within the column (*p* ≤ 0.05). *L* * (*L* = 0 − black, *L* = 100 − white), *a* * (−*a* = green, +*a* = red), *b* * (−*b* = blue, +*b* = yellow).

**Table 2:** Profile of fatty acids in microcapsules and pure camelina oil.

Fatty Acids	Inulin	Pectin	β-Glucan	Gum Arabic	Oil
	Percentage of Fatty Acid [%]				
Palmitc (C16:0)	5.34 ± 0.06 ^c^	5.28 ± 0.02 ^ab^	5.29 ± 0.01 ^bc^	5.28 ± 0.02 ^ab^	5.23 ± 0.03 ^a^
Stearic (C18:0)	2.38 ± 0.02 ^b^	2.40 ± 0.01 ^b^	2.38 ± 0.06 ^b^	2.39 ± 0.07 ^b^	2.33 ± 0.01 ^a^
Oleic (C18:1 n9c)	16.59 ± 0.03 ^d^	16.02 ± 0.01 ^bc^	16.15 ± 0.01 ^c^	15.84 ± 0.03 ^ab^	15.58 ± 0.01 ^a^
α-linolenic (C18:3 n3)	34.05 ± 0.03 ^a^	34.17 ± 0.09 ^a^	34.17 ± 0.07 ^a^	34.25 ± 0.06 ^a^	34.73 ± 0.01 ^b^
Linolenic (C18:2 n6c)	17.82 ± 0.07 ^b^	17.92 ± 0.06 ^a^	17.95 ± 0.02 ^a^	17.96 ± 0.01 ^a^	18.07 ± 0.02 ^c^
Eicosanoic (C20:1 n9)	14.33 ± 0.01 ^a^	14.54 ± 0.01 ^c^	14.36 ± 0.08 ^ab^	14.49 ± 0.02 ^bc^	14.31 ± 0.01 ^a^
Erucic (C22:1 n9)	3.16 ± 0.05 ^abc^	3.21 ± 0.04 ^c^	3.15 ± 0.02 ^ab^	3.18 ± 0.08 ^bc^	3.12 ± 0.01 ^a^
Nervonic (C24:1 n9)	1.03 ± 0.02 ^a^	1.09 ± 0.01 ^a^	1.07 ± 0.02 ^a^	0.54 ± 0.01 ^b^	1.21 ± 0. 04 ^c^

^a, b, c^—letters mean significantly different results within the row (*p* ≤ 0.05).

## References

[1] Bakry A.M., Abbas S., Ali B., Majeed H., Abouelwafa M.Y., Mousa A., Liang L. (2016). Microencapsulation of Oils: A Comprehensive Review of Benefits, Techniques, and Applications. Compr. Rev. Food Sci. Food Saf..

[2] Poshardi A., Aparna K. (2010). Microencapsulation technology: A review. J. Res. ANGRAU.

[3] Singh M., Dua J.S., Menra M., Soni M., Prasad D.N. (2016). Microencapsulation and its various aspects: A review. Int. J. Adv. Res..

[4] Abramovič H., Abram V. (2005). Physico-Chemical Properties, Composition and Oxidative Stability of Camelina sativa Oil. Food Technol. Biotechnol..

[5] O’Dwyer S.P., O’Beirne D., Eidhin D.N., O’Kennedy B.T. (2013). Effects of emulsification and microencapsulation on the oxidative stability of camelina and sunflowers oils. J. Microencapsul..

[6] Belayneh H.D., Wehling R.L., Zhang Y., Ciftci O.N. (2017). Development of omega-3-rich Camelina sativa seed oil emulsions. Food Sci. Nutr..

[7] Mudgil D., Barak S. (2013). Composition, properties and health benefits of indigestible carbohydrate polymers as dietary fiber: A review. Int. J. Biol. Macromol..

[8] Kurek M.A., Karp S., Stelmasiak A., Pieczykolan E., Juszczyk K., Rieder A. (2018). Effect of natural flocculants on purity and properties of β-glucan extracted from barley and oat. Carbohydr. Polym..

[9] Desplaques S., Renou F., Grisel M., Malhiac C. (2012). Impact of chemical composition of xanthan and acacia gums on the emulsification and stability of oil-in-water emulsions. Food Hydrocoll..

[10] Piwińska M., Wyrwisz J., Kurek M., Wierzbicka A. (2015). Hydration and physical properties of vacuum-dried durum wheat semolina pasta with high-fiber oat powder. LWT-Food Sci. Technol..

[11] Shantha N.C., Decker E.A. (1994). Rapid, sensitive, iron-based spectrophotometric methods for determination of perorlride values of food lipids. J. AOAC Int..

[12] Al-Shannaq R., Farid M., Al-Muhtaseb S., Kurdi J. (2015). Emulsion stability and cross-linking of PMMA microcapsules containing phase change materials. Sol. Energy Mater. Sol. Cells.

[13] Temelli F. (1997). Extraction and Functional Properties of Barley β-Glucan as Affected by Temperature and pH. J. Food Sci..

[14] Böger B.R., Georgetti S.R., Kurozawa L.E. (2018). Microencapsulation of grapeseed oil by spray drying. Food Sci. Technol..

[15] Li J., Xiong S., Wang F., Regenstein J.M., Liu R. (2015). Optimization of Microencapsulation of Fish Oil with Gum Arabic/Casein/Beta-Cyclodextrin Mixtures by Spray Drying. J. Food Sci..

[16] Ahmad M., Ashraf B., Gani A., Gani A. (2018). Microencapsulation of saffron anthocyanins using β-glucan and β-cyclodextrin: Microencapsule characterization, release behaviour and antioxidant potential during in-vitro digestion. Int. J. Biol. Macromol..

[17] Fernandes R.V.D.B., Borges S.V., Botrel D.A. (2014). Gum Arabic/starch/maltodextrin/inulin as wall materials on the microencapsulation of rosemary essential oil. Carbohydr. Polym..

[18] Park J.-S., Lim Y.-M., Baik J., Jeong J.-O., An S.-J., Jeong S.-I., Gwon H.-J., Khil M.-S. (2018). Preparation and evaluation of β-glucan hydrogel prepared by the radiation technique for drug carrier application. Int. J. Biol. Macromol..

[19] Chew S.C., Tan C.P., Nyam K.L. (2018). Microencapsulation of refined kenaf (*Hibiscus cannabinus L*.) seed oil by spray drying using β-cyclodextrin/ / sodium caseinate. J. Food Eng..

[20] Kurek M.A., Moczkowska M., Pieczykolan E., Sobieralska M. (2018). Barley β-d-glucan–modified starch complex as potential encapsulation agent for fish oil. Int. J. Biol. Macromol..

[21] Fernandes R.V.D.B., Borges S.V., Botrel D.A., Silva E.K., Costa J.M.G.D., Queiroz F. (2013). Microencapsulation of rosemary essential oil: Characterization of particles. Dry. Technol..

[22] Jafari S.M., Mahdavee Khazaei K., Ghorbani M., Hemmati Kakhki A. (2014). Application of maltodextrin and gum Arabic in microencapsulation of saffron petal’s anthocyanins and evaluating their storage stability and color. Carbohydr. Polym..

[23] Eidhin D.N., Burke J., O’Beirne D. (2003). Oxidative Stability of omega-3-rich Camelina Oil and Camelina Oil-based Spread Compared with Plant and Fish Oils and Sunflower Spread. J. Food Sci..

[24] Charuwat P., Boardman G., Bott C., Novak J.T. (2018). Thermal degradation of long-chain fatty acids. Water Environ. Res..

[25] Fernandes R.V.D.B., Botrel D.A., Silva E.K., Borges S.V., de Oliveira C.R., Yoshida M.I., de Andrade Feitosa J.P., de Paula R.C.M. (2016). Cashew gum and inulin: New alternative for ginger essential oil microencapsulation. Carbohydr. Polym..

